# Characterization and Clinical Utility of *BRAF*^V600^ Mutation Detection Using Cell-Free DNA in Patients with Advanced Melanoma

**DOI:** 10.3390/cancers13143591

**Published:** 2021-07-17

**Authors:** Piotr Rutkowski, Patrick Pauwels, Joseph Kerger, Bart Jacobs, Geert Maertens, Valerie Gadeyne, Anne Thielemans, Katrien de Backer, Bart Neyns

**Affiliations:** 1 Maria Sklodowska-Curie National Research Institute of Oncology, 00-001 Warsaw, Poland; piotr.rutkowski@pib-nio.pl; 2 Department of Pathology, Antwerp University Hospital, 2650 Edegem, Belgium; patrick.pauwels@uza.be; 3 Centre for Oncological Research (CORE), Antwerp University, 2610 Wilrijk, Belgium; 4 Institut Jules Bordet, Université Libre de Bruxelles (ULB), 1000 Brussels, Belgium; joseph.kerger@bordet.be; 5 Biocartis, 2800 Mechelen, Belgium; bart.jacobs@novartis.com (B.J.); gmaertens@biocartis.com (G.M.); 6 F. Hoffmann–La Roche, 4070 Basel, Switzerland; valerie.gadeyne@roche.com (V.G.); anne.thielemans@contractors.roche.com (A.T.); katrien.de_backer@contractors.roche.com (K.d.B.); 7 Department of Medical Oncology, Vrije Universiteit Brussel (VUB), Universitair Ziekenhuis Brussel (UZ Brussel), 1090 Brussels, Belgium

**Keywords:** *BRAF*^V600^ mutations, melanoma, cell-free DNA

## Abstract

**Simple Summary:**

The choice of cancer drug(s) for the treatment of advanced melanoma is based on the types of gene alterations that are present in the patient’s tumor(s). Sometimes, the tumor sample that is obtained from surgery may be degraded, and the test does not provide a reliable result, leading to the selection of the wrong treatment, and, consequently, poor outcomes for the patient. Surgery to obtain fresh tumor samples is inconvenient. In recent years, scientists have learned that fragments of genes from dying cells, including tumors, are constantly being released into the blood. This study shows that the presence of altered genes can be reliably determined using easy-to-obtain blood samples. The study also shows that, while there is a small rate of error with the commonly used tests based on the tumor tissue sample, retests using blood samples may be a less invasive and rapid alternative for identifying the *BRAF* mutation status and selecting the right treatment for these patients.

**Abstract:**

Tissue-based tests for *BRAF*^V600^ mutation-positive melanoma involve invasive biopsy procedures, and can lead to an erroneous diagnosis when the tumor samples degrade. Herein, we explored a minimally invasive, cell-free deoxyribonucleic acid (cfDNA)-based platform, to retest patients for *BRAF*^V600^ mutations. This phase 2 study enrolled adult patients with unresectable/metastatic melanoma. A prescreening testing phase evaluated the concordance between a prior tissue-based *BRAF*^V600^ mutation test result and a subsequent plasma cfDNA-based test result. A treatment phase evaluated the patients who were confirmed as *BRAF*^V600^ mutation-positive, and were treated with cobimetinib plus vemurafenib. It was found that 35/54 patients (64.8%) with a mutant *BRAF* status by prior tissue test had a positive *BRAF*^V600^ mutation with the cfDNA test. Further, 7/118 patients (5.9%) with a wild-type *BRAF* status had a positive *BRAF*^V600^ mutation cfDNA test; tissue retests on archival samples confirmed *BRAF*^V600^ mutation positivity in 5/7 patients (71.4%). One of these patients received BRAF pathway-targeted therapy (cobimetinib plus vemurafenib), and had progression-free survival commensurate with previous experience. In the overall cobimetinib plus vemurafenib-treated population, 29/36 patients (80.6%) had an objective response. The median progression-free survival was 13.6 months (95% confidence interval, 9.5–16.5). Cell-free DNA–based tests may be a fast and convenient option to identify *BRAF* mutation status in melanoma patients, and help inform treatment decisions.

## 1. Introduction

Approximately half of all patients with cutaneous melanoma harbor *BRAF* mutations [1,2,3,4]. *BRAF* pathway-targeted therapies are an optimal treatment option among patients with advanced disease [5,6], and also as an adjuvant treatment for high-risk patients [7,8,9]. The eligibility for BRAF pathway-targeted therapy is predicated on the detection of a *BRAF*^V600^ mutation in the tumor [10,11,12]. Currently, multiple diagnostic methodologies are used to detect the presence of *BRAF* mutations in tumor tissue, including high-resolution melt polymerase chain reaction (PCR); the Cobas 4800 BRAF V600 mutation test (Roche Diagnostics, Indianapolis, IN, USA); real-time allele-specific amplification PCR; next-generation sequencing; digital droplet PCR; immunohistochemistry, which is restricted to the *BRAF*^V600E^ mutation; and Idylla, an automated, PCR-based, molecular platform (Biocartis, Jersey City, NJ, USA) [1,11,13,14,15]. All of these techniques have varying degrees of inherent sensitivity and specificity [1,2,16].

Sample quality is a key factor underlying the reliability of the *BRAF* mutation tests [17]. Traditionally, the samples used for the detection of *BRAF* mutations in patients with melanoma, are formalin-fixed paraffin-embedded tissue biopsies [17,18]. The inherent limitations of formalin-fixed paraffin-embedded tissue samples are at least two-fold. First, the presence of formaldehyde degrades nucleic acids and denatures protein epitopes, which reduces the detectable signal in the sample [19]. Second, discrepancies have been reported between the analysis of primary versus metastatic lesions, especially when there has been a long interval between both the diagnoses [20]. Both the preceding situations can lead to false-negative results that are potentially deleterious for the patient, because they would lead to the selection of the wrong treatment. The other obvious challenge with tumor tissue biopsies is that they constitute an invasive procedure that cannot always be safely repeated. One approach to overcome the limitation of a tissue biopsy-based approach is to use circulating plasma DNA as the test sample [21]. Small fragments of DNA from tumor cells are constantly being released into the lymph and blood. Therefore, the circulating cell-free DNA (cfDNA) has tumor DNA that is representative of all the lesions present in an individual patient. Further, blood sampling is far less invasive procedure than tumor biopsy, and can be easily repeated in an outpatient setting [21]. Thus, plasma cfDNA-based tests could allow for easy rechecking of the *BRAF* mutation status in patients with advanced melanoma and correct diagnoses.

Accordingly, we designed a multicenter phase 2 study to assess the ability of the Idylla platform to detect the presence of *BRAF*^V600^ mutations in cfDNA in the plasma of patients with advanced melanoma, and to determine the clinical benefit derived from the subsequent test-informed treatment decision.

## 2. Materials and Methods

### 2.1. Study Design

This single-arm, open-label, multicenter, phase 2 study enrolled adult patients with unresectable or metastatic melanoma. The study comprised two sequential phases. The first, the prescreening testing phase, provided a preliminary estimate of the concordance between a prior tissue-based *BRAF*^V600^ mutation test result and a subsequent plasma cfDNA-based test result. The second, the treatment phase, evaluated clinical outcomes in all patients confirmed to have *BRAF*^V600^ mutation-positive tumors (either by a prior tissue test, or confirmed by tissue retest for patients who had a positive plasma cfDNA test result following a prior negative tissue test). Within the treatment phase, a substudy (*n* = 8), to evaluate the correlation between the duration of response (DOR), progression-free survival (PFS), and concentration of the *BRAF*^V600^ mutation in patient plasma, was also conducted. To accomplish this substudy evaluation, additional plasma cfDNA tests were performed on plasma samples that were collected prior to the first treatment dose of cobimetinib and vemurafenib, on day 15 of the first cycle, prior to the first dose of each subsequent treatment cycle, and upon disease progression.

### 2.2. Patients

Adult patients with a diagnosis of stage IIIC unresectable locally advanced or stage IV metastatic cutaneous melanoma (as per American Joint Committee on Cancer 7th Edition TNM Staging System) [22], and a tissue test result for the *BRAF*^V600^ mutation, were eligible to enroll in the prescreening phase. The patients entering the treatment phase could have received prior systemic treatment for metastatic melanoma—with the exception of prior BRAF/MEK pathway-inhibitor treatment. Any adverse events (AEs) from prior therapy should have been resolved or be of ≤grade 1 severity per the common terminology criteria for adverse events, version 4.03 [23]. Additional eligibility criteria for the treatment phase included an Eastern Cooperative Oncology Group performance status of 0–2 and adequate renal, hepatic, and end-organ function (see Supplementary Materials Appendix for full eligibility criteria).

### 2.3. Samples and Mutation Analysis

Prior to entering the prescreening phase of the study, the patients had documented *BRAF*^V600^ mutation testing performed by Cobas^®^ or routine PCR testing. Once enrolled, patient plasma was prepared from 10 mL of venous blood collected in an EDTA (ethylenediaminetetraacetic acid) tube for cfDNA analysis using the Idylla ctBRAF mutation assay on the Idylla platform (see Supplementary Materials for additional details) [15,20].

**Figure 1:**
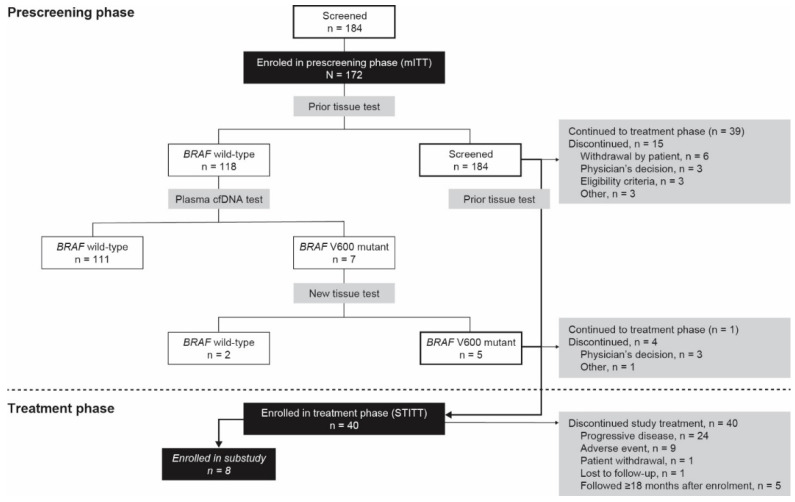
Patient disposition. mITT, modified intention-to-treat; STITT, study treatment intention-to-treat.

For patients with a prior *BRAF* wild-type tissue test result and for whom a *BRAF*^V600^ mutation was detected using plasma cfDNA, tissue samples were obtained to perform a new tissue analysis. If tissue material from the prior test was still available, it was retested using the Idylla platform, by means of the Idylla BRAF mutation assay. If tissue was not available, but it was possible to obtain a new tissue sample from a recent metastasis, this was tested using the Idylla platform. If it was not possible to obtain a new tissue sample from a recent metastasis, the patient was discontinued from the study. To assess the concentration of *BRAF*^V600^ and *NRAS* mutant cfDNA in samples collected during treatment, an Idylla ctNRAS-BRAF mutation assay was performed retrospectively once all samples for each individual patient included in the substudy were collected (Figure 1).

All the patients who satisfied all the inclusion criteria for the prescreening testing phase of the study were also tested for the presence of the *NRAS* mutation using plasma cfDNA. This *NRAS*/*BRAF* mutation testing on serial liquid biopsies was performed as an exploratory post hoc analysis; the results of these analyses did not influence treatment decisions.

### 2.4. Treatments

The patients with tumors that were *BRAF*^V600^ mutation-positive, based on a tumor tissue sample, received oral cobimetinib 60 mg once daily for 21 days, followed by a 7-day rest period in each 28-day cycle and oral vemurafenib 960 mg twice daily. The eligible patients were treated until disease progression as per investigator assessment, unacceptable toxicity, or consent withdrawal—whichever occurred first.

### 2.5. Outcomes and Assessments

Medical history obtained within 28 days of cycle 1 day 1, included confirmation of melanoma, clinically significant diseases within the previous 3 years, major surgeries, and cancer history. The progression of disease during screening was documented.

All measurable and non-measurable lesions were documented at screening, within 28 days prior to cycle 1 day 1. On-treatment tumor assessments were performed every 8 weeks until investigator-determined progression or death, and evaluation of tumor response was conducted—conforming to response evaluation criteria in solid tumors, version 1.1. All patients had a brain screening by computed tomography or magnetic resonance imaging within 28 days prior to cycle 1 day 1, to assess for brain metastases, and subsequently as per local standard of care and as clinically indicated.

Efficacy assessments included objective response rates as per RECIST version 1.1 and Kaplan–Meier estimates for DOR, PFS, and overall survival. For the substudy, the association of these efficacy endpoints with the level of *BRAF* mutation was analyzed.

Safety assessments consisted of monitoring and recording AEs, including serious AEs and nonserious AEs of special interest, performing protocol-specified safety laboratory assessments, measuring protocol-specified vital signs, and conducting other protocol-specified tests that were deemed critical to the safety evaluation of the study. Adverse event severity was assessed using common terminology criteria for adverse events (CTCAE), version 4.03.

### 2.6. Statistical Considerations

Testing of the *BRAF*^V600^ mutation using plasma cfDNA was considered to be of clinical interest if ≥10% of the patient tumors designated as *BRAF* wild-type based on a prior tissue test were subsequently shown to be *BRAF*^V600^ mutation-positive based on cfDNA and confirmed in a new tissue sample or retest of the archival tissue using the Idylla platform. The plasma test was considered not to be of clinical value if ≤3% of *BRAF* wild-type tumors were reclassified as *BRAF*^V600^ mutation-positive tumors.

Based on a Fleming one-stage design with a one-sided α of 0.05 and a power of 0.90, a sample size of ≥104 patients for whom the prior tissue test indicates wild-type status should be identified. Assuming an equal number of patients with *BRAF*^V600^ mutation and wild-type *BRAF* mutation based on the prior tissue test, a total of 208 patients were to be enrolled in the prescreening phase of the study. For 104 patients, the power to show that the frequency of reclassifying tumors as *BRAF*^V600^ mutation-positive exceeds 3% is 84%, 75%, and 63% for frequencies of interest of 9%, 8%, and 7%, respectively.

The modified intention-to-treat (mITT) population comprised all enrolled patients with a documented *BRAF*^V600^ tissue test result on melanoma tumor tissue at study entry and an available result of the plasma cfDNA test. The study treatment intention-to-treat (STITT) population comprised all enrolled patients with a plasma cfDNA test who received ≥1 dose of cobimetinib or vemurafenib. The analysis of the primary objective was performed on all patients in the mITT population with *BRAF*^V600^ wild-type status based on a prior tissue test.

The analysis of the secondary and the exploratory objectives was performed on all patients in the mITT population. The analysis of the secondary objectives related to clinical outcome (tumor response) was performed on the STITT population. Patients in the substudy were a subset of patients from the STITT population.

## 3. Results

### 3.1. Patient Disposition

A total of 184 patients consented, and were evaluated for eligibility for the prescreening phase. Of these patients, 10 did not have documented *BRAF*^V600^ mutation test results on melanoma tissue upon study entry, and two had no plasma cfDNA test results (Figure 1). The remaining 172 patients represented the mITT population. Of these, 118 (68.6%) had a wild-type *BRAF* status and 54 (31.4%) had a mutant *BRAF*^V600^ status. Among the 118 patients with an initial wild-type *BRAF* status, the tumors in five of these patients were confirmed to be *BRAF*^V600^ mutant in the tissue retests, and one patient continued into the treatment phase. Of the 54 patients with a mutant *BRAF*^V600^ status, based on the prior tissue test, 39 continued into the treatment phase. The baseline characteristics of the patients are shown in Table 1.

### 3.2. Prescreening Phase

#### 3.2.1. Primary Endpoint

Among the 118 patients with a wild-type *BRAF* status, seven (5.9%) had a positive *BRAF*^V600^ mutation test result, based on the plasma cfDNA test. Tissue retests on archival samples confirmed the *BRAF*^V600^ mutation in five (71.4%) of the seven patients. Thus, for 4.2% of the patients with a wild-type *BRAF* status, the presence of a *BRAF*^V600^ mutation was confirmed in a tissue retest; the one-sided test, comparing this frequency with the a priori hypothesis of 3%, was not statistically significant (*p* = 0.215).

#### 3.2.2. Secondary Endpoint

The plasma cfDNA test was concordant with the prior tissue test in 35 of 54 of the patients who were classified as *BRAF*^V600^ mutation-positive, and in 111 of 118 of the patients with a wild-type status (Table 2). Relative to tissue testing, the plasma test demonstrated 64.8% sensitivity and 94.1% specificity. Of the 42 patients with a *BRAF*^V600^ mutant plasma cfDNA test result, 35 had a mutant status, based on the prior tissue test. The positive predictive value (i.e., the likelihood that the mutation detected with the plasma test corresponds to the tissue test) was 83.3%. Of the 130 patients with a wild-type plasma test result, 111 had a wild-type status, based on the prior tissue test. The negative predictive value (i.e., the likelihood that the wild-type status detected with the plasma test corresponds to the tissue test) was 85.4%.

#### 3.2.3. Exploratory Endpoint

Of the 172 patients in the mITT population, 171 (99.4%) had an available *NRAS* mutation test result. Of these patients, 149 (87.1%) had an *NRAS* wild-type status and 22 (12.9%) had a mutant status. A summary of the relationship of *NRAS* mutant status and *BRAF*^V600^ mutant plasma test results is presented in Table 2. The mutations in *BRAF* and *NRAS* were mutually exclusive.

### 3.3. Treatment Phase

#### 3.3.1. Efficacy Outcomes

In the STITT population, 29 of the 36 patients (80.6%) with target lesions and measurable disease, had either a complete response (three patients) or a partial response (26 patients) (Table 3). One patient (2.8%) had progressive disease, and two patients (5.6%) had stable disease. The median PFS (95% confidence interval (CI)) was 13.6 months (9.5–16.5). Of the 29 patients who had a complete or partial response, the median DOR (95% CI) was 11.0 months (9.2—not estimable). The median overall survival time was not estimable at the time of the analysis.

#### 3.3.2. Correlation of Mutation Testing and Clinical Outcome

The *BRAF*^V600^ mutation was detected in the plasma at prescreening for 26 of the 40 treated patients. The median quantitation cycle (Cq) for the *BRAF* mutation was 40.25. The median PFS was 12.8 months and 10.8 months for the patients below and above the median mutation Cq, respectively; however, the Cq was not significantly associated with PFS duration (*p* = 0.653). Of these 26 patients, 22 had a complete or partial response. No significant relationship was found between mutation Cq and the DOR (*p* = 0.409). The median DOR was similar for these patients, regardless of whether the mutation Cq was above or below the median (10.7 vs. 10.9 months, respectively).

The clinical outcome was analyzed separately for patients with a *BRAF*^V600^ mutation, based on detection of the mutation in the plasma test at the prescreening. The median PFS was 14.8 months for the patients with a *BRAF* wild-type plasma test result, and 12.8 months for the patients with a *BRAF*^V600^ mutation plasma test result (*p* = 0.286). The median DOR was not evaluable for the patients with a *BRAF* wild-type plasma test result, and was 10.9 months for the patients with a *BRAF*^V600^ mutation plasma test result (*p* = 0.220).

#### 3.3.3. Substudy

The concentration of the *BRAF*^V600^ mutation before the first dose was evaluated as a prognostic factor/metric for PFS duration in the eight patients who were participating in the substudy. The *BRAF*^V600^ mutation was observed at the start of cycle 1 for five of the eight patients (62.5%). The frequency of detection of the *BRAF*^V600^ mutation then decreased to 28.6% at the last assessment. All of these mutations were *BRAF*^V600E/E2/D^, except for one instance of *BRAF*^V600K/R/W^. No correlation between the plasma concentration of the mutation (average Cq for mutation) at the start of cycle 1, and the PFS duration, was observed (Spearman’s rank correlation coefficient: −0.100; *p* = 0.873).

#### 3.3.4. Exposure and Safety

The mean duration of the treatment with both cobimetinib and vemurafenib was 11.2 ± 9.6 months (median, 7.9 months; range, 0.4–36.8 months). The median number of cycles was 9.0 for both cobimetinib and vemurafenib. The most frequently reported AEs were rash (47.5%), blood creatinine phosphokinase level increase (32.5%), diarrhea (32.5%), photosensitivity reaction (27.5%), pyrexia (27.5%), arthralgia (27.5%), and maculopapular rash (22.5%) (Table 4).

## 4. Discussion

The current study aimed to evaluate the clinical utility of a plasma-derived cfDNA test for the detection of *BRAF*^V600^ mutations in patients with metastatic melanoma, in whom the original testing using tumor biopsy falsely diagnosed the *BRAF*^V600^ wild-type status. It was observed that ~6% of the patients who were originally classified as having *BRAF* wild-type tumors tested positive for *BRAF* mutations using the cfDNA test. The sensitivity of the plasma cfDNA testing was 64.8%, and its positive and negative predictive values were 83.3% and 85.4%, respectively. No significant difference was observed between the median PFS and the median DOR for the patients with a *BRAF* wild-type versus *BRAF*^V600^ mutation-positive test result using cfDNA testing.

Previous studies have shown that cfDNA testing may be a surrogate for determining *BRAF* mutations in patients with melanoma [24,25,26,27,28]. In our study, the presence of *BRAF* mutations was confirmed by a subsequent tissue-based retest in five of the seven patients who showed *BRAF* mutations using cfDNA testing. The remaining two patients showed *BRAF* wild-type tumors upon the retest, which could potentially be a result of the archival tissue sample being degraded. Although the study did not meet its predefined criteria for the clinical significance of cfDNA testing (≥10% of patients designated as *BRAF* wild-type based on a prior tissue test to subsequently show *BRAF*^V600^ mutations on cfDNA testing), ~6% of *BRAF* wild-type patients were subsequently shown to be *BRAF*^V600^-mutation-positive, based on cfDNA tests, suggesting that cfDNA testing may have a place in the clinical setting. Furthermore, one of the five *BRAF* mutation-positive patients went on to receive *BRAF*-targeted therapy and responded to treatment with a PFS that was in a range that was observed in previous experience [29]. These results suggest that cfDNA testing may be a quick and easy alternative to identify *BRAF* mutations in patients for whom an archival tumor tissue sample may not be available in sufficient quality to allow for a retesting population, so they can benefit from the early initiation of *BRAF*-targeted therapy. However, it should be noted that cfDNA testing demonstrated a sensitivity of 64.8% in the present study. Therefore, although the cfDNA technique may be beneficial in the initial screening for the *BRAF* mutation status (owing to the ease of obtaining a blood sample versus a tumor re-biopsy, and the possibility of loss of the archival sample due to degradation, as seen in two patients in this study), retesting of the archival tumor tissue, if available, should be the primary choice for confirmation of the *BRAF* mutation status.

This study also evaluated any potential correlation with the cell-free *BRAF* mutation load of the plasma and treatment outcomes, as suggested by previous reports [29,30,31]; however, no appreciable differences were observed in either the median PFS or DOR among patients in whom the Cq for the *BRAF* mutation was above or below the median Cq of 40.25. The substudy, which tested *BRAF* mutation load as a metric of treatment response, showed that there was a decrease in the frequency of detection of the *BRAF*^V600^ mutation during the treatment, and the assay failed to detect the *BRAF*^V600^ mutation in a number of patients as the treatment progressed. Overall, the treatment outcomes and the observed safety profile with combination treatment with cobimetinib plus vemurafenib, were consistent with the pivotal phase 3 data in a population of patients with advanced melanoma and *BRAF* mutations [29,32].

A key limitation of the study was the relatively small number of patients who were included. In addition, only one patient whose tumor was originally classified as *BRAF* wild-type, and subsequently flagged as *BRAF* mutation-positive by the cell-free assay, went on to receive and benefit from targeted therapy with cobimetinib plus vemurafenib. The strengths of the study include its prospective nature, and that consecutive eligible and consenting patients at the study centers were enrolled (i.e., there was no selection bias). Furthermore, the study confirmed that a high percentage of patients that are designated as having *BRAF*-mutated tumors, using tissue-based tests, have positive results in cfDNA testing.

## 5. Conclusions

The study shows that plasma testing using cfDNA is a less invasive and rapid alternative to confirm the presence of *BRAF* mutations versus tumor biopsies, and may help in the early identification of patients who are more likely to benefit from treatment with *BRAF* inhibitors. However, cfDNA testing should be used with caution in patients undergoing *BRAF* inhibitor therapy, owing to its decreased sensitivity with treatment in these patients. No significant correlation between the use of cfDNA testing, and PFS and DOR could be determined among the patients receiving cobimetinib plus vemurafenib treatment. Additional studies in a larger patient population are warranted to confirm these results.

## Figures and Tables

**Table 1:** Baseline demographic, disease, biopsy, and mutation characteristics.

	mITT	STITT
	*n* = 172	*n* = 40
Age, median years (range)	62.5 (20–93)	56.5 (26–82)
Sex, *n* (%)		
Male	84 (48.8)	18 (45.0)
Female	88 (51.2)	22 (55.0)
ECOG score, *n* (%)		
0	NE	21 (52.5)
1	NE	16 (40.0)
2	NE	3 (7.5)
Time since diagnosis of metastases, median months (range)	19.3 (0.1–260.7)	12.4 (0.8–260.7)
Age at diagnosis of metastases, median years (range)	59.1 (18–91)	53.5 (26–83)
Disease stage at study entry, *n* (%)		
Unresectable stage IIIC	16 (9.3)	1 (2.5)
Stage IV	156 (90.7)	29 (97.5)
Measurable disease at study entry, *n* (%)	147 (85.5)	37 (92.5)
Number of target lesions, *n* (%)		
0–3	NE	22 (55.0)
>3	NE	18 (45.0)
Type of tissue material, *n* (%)		
Archival	140 (81.4)	30 (75.0)
Recent	32 (18.6)	10 (25.0)
Prior tissue *BRAF* mutation test result, *n* (%)		
*BRAF* wild-type	118 (68.6)	1 (2.5)
*BRAF*^V600^ mutation	54 (31.4)	39 (97.5)
Prior therapy, *n* (%)		
Immunotherapy	–	9 (33)
Targeted therapy	–	0
Other systemic therapy	–	7 (17.5)
Investigational treatment	–	1 (2.5)
Radiotherapy	–	8 (20.0)

ECOG, Eastern Cooperative Oncology Group; mITT, modified intention-to-treat; NE, not evaluated; STITT, study treatment intention-to-treat.

**Table 2:** Comparison of tissue-based and cfDNA-based mutation tests. (**A**) Comparison of plasma and tissue *BRAF*^V600^ mutation test results, and (**B**) comparison of *BRAF* and *NRAS* plasma mutation tests results in the mITT population. Grey-shaded cells designate the discordance between the respective tests. cfDNA, cell-free DNA; mITT, modified intention-to-treat.

A. BRAF		Plasma cfDNA Test Result		
		Wild-Type	Mutant	
**Tissue test result**	Wild-type	111	7	Patients with wild-type tissue test = 118
	Mutant	19	35	Patients with mutant tissue test = 54
		Patients with wild-type plasma test = 130	Patients with mutant plasma test = 42	
**B. Plasma**		**BRAF cfDNA test result**		
		**Wild-type**	**Mutant**	
**NRAS cfDNA test result**	Wild-type	107	42	Patients with wild-type NRAS = 149
	Mutant	22	0	Patients with mutant NRAS = 22
		Patients with wild-type plasma test = 129	Patients with mutant plasma test = 42	

**Table 3:** Objective response rate and PFS (STITT).

	STITT *n* = 40	Archival Tissue * n = 29	Recent Tissue ^†^ *n* = 10
Objective response rate, ‡ *n* (%)			
CR	3 (8.3)	3 (11.5)	–
PR	26 (72.2)	20 (76.9)	6 (60.0)
SD	2 (5.6)	–	2 (20.0)
PD	1 (2.8)	–	1 (10.0)
Other nonresponders	4 (11.1)	3 (11.5)	1 (10.0)
Responders (CR + PR), *n*/*n*′ (%)	29/36 (80.6)	23/26 (88.5)	6/10 (60.0)
95% CI	64.0–91.8	69.8–97.6	26.2–87.8
Duration of Response, *n*′ ‡	29	23	*n*′ = 6
Median, months (95% CI)	11.0 (9.2–NE)	11.0 (9.2–NE)	8.8 (3.6–NE)
Progression-free survival			
Median months (95% CI)	13.6 (9.5–16.5)	14.5 (10.8–NE)	6.2 (3.6–NE)

CI, confidence interval; CR, complete response; *n*′, number of patients considered in the analysis; percentages calculated based on *n*′; NE, not estimable; PD, progressive disease; PR, partial response; SD, stable disease; STITT, study treatment intention-to-treat. * Patients for whom treatment was based on the mutation identified by prior tissue test and for whom archival tissue was used. ^†^ Patients for whom treatment was based on the mutation identified by prior tissue test and for whom recent tissue was used. ^‡^ For patients with measurable disease at screening having responded during the study (CR or PR).

**Table 4:** Common treatment-emergent adverse events (incidence ≥10% by preferred term).

System Organ Class/Preferred Term	*n* = 40
Any treatment-emergent adverse event, *n* (%)	39 (97.5)
Skin and subcutaneous tissue disorders, *n* (%)	33 (82.5)
Rash	19 (47.5)
Photosensitivity reaction	11 (27.5)
Maculopapular rash	9 (22.5)
Alopecia	4 (10.0)
Investigations, *n* (%)	22 (55.0)
Blood creatinine phosphokinase increased	13 (32.5)
Gamma-glutamyltransferase increased	5 (12.5)
C-reactive protein increased	4 (10.0)
General disorders and administration site conditions, *n* (%)	21 (52.5)
Pyrexia	11 (27.5)
Fatigue	8 (20.0)
Oedema peripheral	5 (12.5)
Infections and infestations, *n* (%)	21 (52.5)
Upper respiratory tract infection	8 (20.0)
Conjunctivitis	6 (15.0)
Urinary tract infection	4 (10.0)
Gastrointestinal disorders, *n* (%)	20 (50.0)
Diarrhea	13 (32.5)
Vomiting	6 (15.0)
Nausea	5 (12.5)
Musculoskeletal and connective tissue disorders, *n* (%)	14 (35.0)
Arthralgia	11 (27.5)
Musculoskeletal pain	4 (10.0)
Myalgia	4 (10.0)
Pain in extremity	4 (10.0)
Eye disorders, *n* (%)	12 (30.0)
Vision blurred	6 (15.0)
Chorioretinopathy	4 (10.0)
Nervous system disorders, *n* (%)	12 (30.0)
Headache	6 (15.0)
Metabolism and nutrition disorders, *n* (%)	7 (17.5)
Decreased appetite	4 (10.0)
Hypokalemia	4 (10.0)
Hypertension	5 (12.5)

## Data Availability

Qualified researchers may request access to individual patient-level data through the clinical study data request platform (https://vivli.org/, accessed on 19 January 2021). Further details on Roche’s criteria for eligible studies are available here (https://vivli.org/members/ourmembers/, accessed on 19 January 2021). For further details on Roche’s global policy on the sharing of clinical information and how to request access to related clinical study documents, see here (https://www.roche.com/research_and_development/who_we_are_how_we_work/clinical_trials/our_commitment_to_data_sharing.htm, accessed on 19 January 2021).

## Supplementary Materials

The following is available online at https://www.mdpi.com/article/10.3390/cancers13143591/s1, Supplementary material appendix: patient eligibility criteria.
