# Supplementary material for: Characterization and Clinical Utility of BRAFV600 Mutation Detection Using Cell-Free DNA in Patients with Advanced Melanoma
Source: Cancers (Basel). 2021 Jul 17;13(14):3591. doi: 10.3390/cancers13143591 (PMC8305047; doi:10.3390/cancers13143591)
Supplement: Supplementary file 1 [file cancers-13-03591-s001.zip › cancers-1247398-supplementary.pdf]

SUPPLEMENTARY MATERIAL APPENDIX

I. Patient Eligibility Criteria

Inclusion Criteria

Patients were eligible for inclusion in the prescreening phase of the study if they met the following inclusion criteria:

1. Adults aged ≥18 years
2. Able to participate and willing to give written informed consent prior to performance of any study-related procedures and to comply with the prescreening phase of the study protocol
3. Patients with histologically confirmed cutaneous melanoma, either unresectable stage IIIc or stage IV metastatic melanoma, as defined by the American Joint Committee on Cancer 7th Edition TNM Staging System (Appendix 5 of the protocol)
4. Documentation of *BRAF* V600 tissue test result on melanoma tumour tissue.

Please note: Patients may or may not have received prior systemic treatment for metastatic melanoma. All adverse events, started during prior systemic treatment, should have either resolved or have been of ≤ grade 1 severity according to Common Terminology Criteria for Adverse Events version 4.03. Patients with measurable or nonmeasurable disease (Response Evaluation Criteria in Solid Tumours version 1.1) were eligible. Patients were eligible for inclusion in the treatment phase of the study if they met the following inclusion criteria:

General inclusion criteria

1. Able to participate and willing to give written informed consent prior to performance of any study-related procedures and to comply with the treatment phase of the study protocol
2. Eastern Cooperative Oncology Group performance status of 0–2
3. Adequate hematologic and end-organ function, defined by the following laboratory results obtained within 14 days prior to first dose of study drug treatment
4. Adequate liver and renal function
   a. Bilirubin value ≤1.5x upper limit of normal (ULN)

b. Aspartate aminotransferase (AST), alanine aminotransferase (ALT), and alkaline phosphatase levels ≤3x ULN with the following exceptions:
    i. Patients with documented liver metastases: AST or ALT level ≤5x ULN
    ii. Patients with documented liver or bone metastases: alkaline phosphatase level ≤5x ULN

c. Serum creatinine level ≤1.5x ULN or creatinine clearance (CrCl) ≥40 mL/min on the basis of measured CrCl from a 24-hour urine collection or Cockcroft-Gault glomerular filtration rate estimation

5. Negative serum pregnancy test prior to commencement of dosing in women of childbearing potential.
6. Absence of any psychological, familial, sociological, or geographical condition that potentially hampers compliance with the study protocol and treatment regimen and follow-up after treatment discontinuation schedule; those conditions should be discussed with the patient before trial entry.
7. Female patients of childbearing potential and male patients with partners of childbearing potential must have agreed to always use two effective forms of contraception during the course of the study and for ≥6 months after completion of study therapy.
    a. Females of childbearing potential are defined as sexually mature women without prior oophorectomy or hysterectomy who have had menses within the last 12 months.
    b. Females are not considered to be of childbearing potential if amenorrhoeic for >12 months but <2 years, with follicle-stimulating hormone (FSH) levels ≥40 IU/L.
    c. Effective forms of contraception include surgical sterilisation, a reliable barrier method with spermicide, birth control pills, or contraceptive hormone implants. Note that potential interactions between vemurafenib and hormonal contraceptives may decrease the effectiveness of hormonal contraception.
8. Patients needed to be able to swallow tablets.
9. Documentation of *BRAF* V600 mutation–positive status in melanoma tumour tissue using a validated tissue test.

Exclusion Criteria

Patients were excluded from entering the treatment phase if they met any of the following exclusion criteria:

1. History of prior RAF or MEK pathway inhibitor treatment.
2. Palliative radiotherapy within 14 days prior to the first dose of study treatment. Use of prior chemotherapy or immunotherapy (including treatment with an anti-PD1, anti-PDL1, or anti–CTLA-4 monoclonal antibody) within 4 weeks before first drug administration.
3. Evidence of retinal pathology on ophthalmologic examination that is considered a risk factor for neurosensory retinal detachment/central serous chorioretinopathy, retinal vein occlusion (RVO), or neovascular macular degeneration.
4. Systemic risk factors for RVO as listed below. Patients were excluded if they had one of the following conditions:
   a. Uncontrolled glaucoma with intraocular pressures ≥21 mmHg
   b. Uncontrolled hypercholesterolemia ≥ grade 2
   c. Hypertriglyceridemia ≥ grade 2
   d. Hyperglycemia ≥ grade 2
5. History of clinically significant cardiac dysfunction, including the following:
   a. Current unstable angina
   b. Symptomatic congestive heart failure of New York Heart Association (Appendix 4 of the protocol) class 2 or higher
   c. History of congenital long QT syndrome or mean (average of triplicate measurements) QTcF ≥450 msec at baseline
   d. Uncontrolled hypertension ≥ grade 2 (patients with a history of hypertension controlled with antihypertensive agents to ≤ grade 1 are eligible)
   e. Left ventricular ejection fraction below institutional lower limit of normal or <50%, whichever is lower

6. Current, severe, and uncontrolled systemic disease
7. Major surgery or traumatic injury within 14 days prior to first dose of study treatment
8. History of malabsorption or other condition that would interfere with absorption of study drugs
9. Pregnant, lactating, or breastfeeding
10. Unwillingness or inability to comply with study and follow-up procedures
11. The following foods and supplements were prohibited ≥7 days prior to initiation of and during study treatment:
    a. St. John's wort or hyperforin (potent cytochrome P450 CYP3A4 enzyme inducer)
    b. Grapefruit juice (potent cytochrome P450 CYP3A4 enzyme inhibitor)

Notes:

- Investigators were to ensure that all study enrolment criteria were met at screening. If a patient's clinical status changed after screening but before the first dose of the study medication was given, such that he or she no longer met all eligibility criteria, then the patient was excluded from participation in the study.
- Retesting of abnormal laboratory values that led to exclusion was allowed once. The investigator could consider the patient eligible if the previous abnormal laboratory test result was within protocol acceptable range on a repeat testing.
